# *Enterococcus* species identified by MALDI-TOF MS in milk from dairy cow mastitis cases and herd surveys

**DOI:** 10.1007/s11259-025-10834-5

**Published:** 2025-07-23

**Authors:** Fernando Ulloa, Martina Penati, Valentina Monistero, Valerio Bronzo, Paolo Moroni, Miguel Salgado, Maria Filippa Addis

**Affiliations:** 1 Escuela de Graduados, Facultad de Ciencias Veterinarias, Universidad Austral de Chile, Valdivia, 5090000 Chile; 2 Facultad de Ciencias Veterinarias, Instituto de Medicina Preventiva Veterinaria, Universidad Austral de Chile, Valdivia, 5090000 Chile; 3 Department of Veterinary Medicine and Animal Science, University of Milan, Lodi, 26900 Italy; 4 Laboratory of Infectious Diseases of Domestic Animals (MiLab), University of Milan, Lodi, 26900 Italy

**Keywords:** Enterococci, Species identification, Clinical mastitis, Subclinical mastitis, Full herd survey

## Abstract

*Enterococcus* species are increasingly recognized as mastitis pathogens in dairy cows. Reliable species information is required for correctly defining the regional epidemiology of enterococcal mastitis, establishing its relationships with management variables, and understanding its impact on udder health. We investigated the species distribution of enterococci in bovine milk from subclinical mastitis (SCM) and clinical mastitis (CM) cases and full herd surveys (HS) using MALDI-TOF MS as identification method. A total of 21,864 milk samples from 106 dairy herds were routinely collected and analyzed according to the National Mastitis Council (NMC) guidelines over one year. *Enterococcus* spp. were found in 4.86% of CM, 5.05% of SCM, and 1.37% of HS milk samples. Overall, *E. saccharolyticus* was the most prevalent species (55.65%), followed by *E. faecium* (27.25%), and *E. cecorum* (5.51%), which showed a significant association with SCM (p-value = 0.0128). This study provides novel data on the *Enterococcus* species distribution in full herds survey and mastitis cases, highlighting the relevance of MALDI-TOF MS in refining enterococcal mastitis epidemiology, as true prevalence and species distribution may have been skewed by conventional biochemical tests.

## Introduction

*Enterococcus* species are Gram-positive, facultative anaerobic bacteria widely distributed as commensals in the gastrointestinal tracts of humans, livestock, and insects (Guzman Prieto et al. 2016). In dairy production, *Enterococcus* spp. is classified within environmental streptococci and streptococci-like organisms (SSLO), alongside *Streptococcus* spp. and *Lactococcus* spp., due to their ubiquity in farm environments (e.g., bedding, soil, and water) and their role as causative agents of intramammary infections (IMI)(Klaas and Zadoks 2018). Globally, enterococci contribute to both clinical mastitis (CM) and subclinical mastitis (SCM), with prevalence varying from 1.3% in Sweden (Duse et al. 2021) to 1.73% in China (Song et al. 2020), up to 2.4% in France (Botrel et al. 2010). Recent data have found *Enterococcus* spp. in 3.49% of SCM and 2.10% of CM samples in Italy (Addis et al. 2024), though these figures derive from genus-level identification. Species-specific differences are critical, as *E. faecium* is associated with multidrug resistance, while *E. faecalis* exhibits virulence traits such as biofilm formation and mammary epithelial cell invasion (Guzman Prieto et al. 2016; Rodrigues et al. 2022). Many species are non-pathogenic, but *E. faecium* and *E. faecalis* are commonly considered opportunistic pathogens implicated in severe infections, including bovine mastitis, and are notorious for their intrinsic and acquired antimicrobial resistance (AMR; (Guimarães et al. 2024). These bacteria exhibit reduced susceptibility to glycopeptides, β-lactams, and fluoroquinolones, and show high resistance levels to aminoglycosides driven by genetic mechanisms such as resistance genes (e.g., *tet*, *erm*) often located on mobile genetic elements and class I integrons (Gao et al. 2019; Liu et al. 2024). Their resistance profiles not only complicate the effective treatment of the infections they cause but also position enterococci as key indicators of antimicrobial resistance dissemination dynamics within dairy farm ecosystems, with significant implications for both animal and public health (Różańska et al. 2019; Juliano et al. 2022).

Accurate species identification with advanced diagnostic methods is crucial for correctly defining enterococcal mastitis epidemiology, its management and environmental drivers, and its impact on udder health. For instance, emerging evidence points to less-studied species as significant mastitis pathogens, such as *E. saccharolyticus*, when identified with Matrix-Assisted Laser Desorption/Ionization Time-of-Flight Mass Spectrometry (MALDI-TOF MS; (Guimarães et al. 2024). Traditional biochemical methods, including API galleries and VITEK 2, have dominated historical studies but frequently misclassified *Enterococcus* spp. as *Streptococcus* or *Lactococcus* due to phenotypic overlap (Werner et al. 2014; Guimarães et al. 2024). Biochemical assays may underestimate species of veterinary interest, like *E. saccharolyticus*, while overreporting *E. faecalis* or *E. faecium* (Różańska et al. 2019; Gao et al. 2019). This diagnostic limitation has skewed epidemiological profiles and hindered targeted management strategies. In contrast, MALDI-TOF MS offers rapid, high-resolution species identification, achieving > 90% accuracy in mastitis pathogen speciation (Nonnemann et al. 2019). Furthermore, a high agreement has been demonstrated between MALDI-TOF MS identification and molecular techniques like gap PCR-RFLP for mastitis-associated bacteria (Rosa et al. 2022). Recent applications of MALDI-TOF MS in dairy contexts have revealed *E. saccharolyticus* as the predominant species in CM milk (62.4%) and *E. faecium* in bulk tank milk (BTM; 67.8%) in Brazil, challenging prior assumptions (Guimarães et al. 2024).

Mastitis control and treatment remain the primary reasons for antimicrobial use in dairy farming, highlighting the need for precise epidemiological data to support prudent antibiotic use (Ruegg 2022). In this context, misidentification of *Enterococcus* species may lead to ineffective management or inappropriate therapies, exacerbate resistance, and overlook emerging pathogens, compromising herd health and milk quality (Cameron et al. 2016). To address this, our study presents the data on *Enterococcus* spp. isolates identified by MALDI-TOF MS and obtained from CM, SCM, and HS bovine milk samples across 106 dairy farms in Northern Italy. By providing a detailed species-level profile, we aimed to refine regional epidemiology, enabling future elucidation of ecological associations and farm-specific interventions for enterococcal mastitis control.

## Materials and methods

### Milk samples and contributing herds

Milk samples were shipped to the Animal Infectious Disease Laboratory (MiLab) at the University of Milan between December 2022 and November 2023 to be processed during the diagnostic routine. The sample set included 106 dairy farms located in Northern Italy, housing Holstein-Friesian cows in free-stall barns. Among these farms, 96 and 34 sent quarter milk samples with clinical mastitis (CM) and subclinical mastitis (SCM), respectively, while 78 sent full herd survey (HS) as composite milk samples. Throughout the collection process, all personnel followed National Mastitis Council (NMC) protocols (Adkins et al. 2017).

For CM, trained personnel identified affected mammary quarters using established clinical criteria (Adkins et al. 2017). The evaluation involved visually inspecting the first milk streams for abnormalities such as flakes, clots, discoloration, or watery consistency, as well as checking for udder swelling, redness, or pain. SCM samples were collected from cows reported by high somatic cell counts (SCC) in recent Dairy Herd Improvement (DHI) records. These cows were further evaluated with the California Mastitis Test (CMT), and milk samples from quarters testing positive for CMT without visible clinical signs were classified as SCM following established guidelines (Adkins et al. 2017). HS milk, representing pooled milk of the four cow quarters, was collected during herd-wide sampling events to determine the prevalence of infectious pathogens at the farm level.

### Bacteriological analysis of milk

Milk samples were processed for bacterial culture in accordance with NMC standards (Adkins et al. 2017), as outlined previously (Addis et al. 2024). In summary, 10 µL of milk was plated on blood agar (Microbiol, Cagliari, Italy), and plates were aerobically incubated at 37 °C for 24–48 h. The plates were then evaluated and categorized as positive, contaminated, or negative based on NMC criteria. From each positive sample, representative colonies were identified using MALDI-TOF MS and the MBT Compass^®^ Library Revision H (2022). Identification scores ≥ 2.0 were considered reliable for species-level identification, while scores ≥ 1.7 and < 2.0 indicated genus-level identification. Isolates scoring < 1.7 were re-analyzed in duplicate, and if subsequent scores remained < 1.7, they were classified as unidentified (Rosa et al. 2022).

### Data analysis

This study analyzed a total of 21,864 milk samples, including 6,278 from CM, 4,039 from SCM, and 11,547 from HS milk. Data regarding farm identification, sampling dates, sample classifications, microbiological results, and identification scores were recorded in a Microsoft Access database (Microsoft Office, version 16.82, 2024). Furthermore, information on subclinical and clinical status was recorded. Data extraction was performed using Microsoft Excel (version 16.82, 2024), utilizing pivot tables and built-in functions for descriptive statistical analysis. Statistical analysis was carried out with SPSS version 29.0 (IBM, SPSS, Armonk, USA). A multinomial logistic regression model was applied to assess the prevalence of *Enterococcus* species in SCM and CM cases, considering the subclinical condition as the reference. Wald statistics were used for parameter estimation, and statistical significance was determined at p-values < 0.05 and < 0.01.

## Results

The bacteriological culture results for the 21,864 milk samples are summarized in Table 1 according to the sample type. Out of 6,278 CM milk samples, 63.57% (3,991) were culture-positive; from these positive cultures, 194 *Enterococcus* spp. isolates (4.86%) were identified. Out of 4,039 SCM milk samples, 69.03% (2,788) were culture-positive, from which 113 *Enterococcus* spp. isolates (4.05%) were identified. Out of 11,547 HS milk samples, 32.37% (3,738) were culture-positive, and from these, 51 *Enterococcus* spp. isolates (1.36%) were identified (Table 2).

**Table 1:** Bacteriological culture results obtained for all the milk samples considered in this study, according to their respective categories

Species	CM quarter milk	SCM quarter milk	HS composite milk	Total general
*Streptococcus* spp.	1,259 (31.55%)	578 (20.73%)	526 (14.07%)	2363 (22.49%)
NASM*	947 (23.73%)	1,162 (41.68%)	1,819 (48.66%)	3,928 (37.38%)
*E. coli*	641 (16.06%)	82 (2.94%)	127 (3.4%)	850 (8.09%)
*Serratia* spp.	230 (5.76%)	142 (5.09%)	42 (1.12%)	414 (3.94%)
*Enterococcus* spp.	194 (4.86%)	113 (4.05%)	51 (1.36%)	358 (3.41%)
*Corynebacterium* spp.	125 (3.13%)	229 (8.21%)	287 (7.68%)	641 (6.1%)
*S. aureus*	109 (2.73%)	198 (7.1%)	160 (4.28%)	467 (4.44%)
*Klebsiella* spp.	74 (1.85%)	23 (0.82%)	39 (1.04%)	136 (1.29%)
Gram negative	70 (1.75%)	32 (1.15%)	184 (4.92%)	286 (2.72%)
Bacilli	53 (1.33%)	8 (0.29%)	27 (0.72%)	88 (0.84%)
Yeast	51 (1.28%)	98 (3.52%)	6 (0.16%)	155 (1.48%)
*Pasteurella multocida*	46 (1.15%)	10 (0.36%)	5 (0.13%)	61 (0.58%)
*Pseudomonas* spp.	44 (1.1%)	14 (0.5%)	21 (0.56%)	79 (0.75%)
*T. pyogenes*	37 (0.93%)	11 (0.39%)	18 (0.48%)	66 (0.63%)
*Enterobacter* spp.	29 (0.73%)	6 (0.22%)	23 (0.62%)	58 (0.55%)
*Prototheca* spp.	23 (0.58%)	4 (0.14%)	202 (5.4%)	229 (2.18%)
*Lactococcus* spp.	21 (0.53%)	39 (1.4%)	52 (1.39%)	112 (1.07%)
*Citrobacter* spp.	14 (0.35%)	6 (0.22%)	7 (0.19%)	27 (0.26%)
*A. viridans*	13 (0.33%)	17 (0.61%)	109 (2.92%)	139 (1.32%)
Other	8 (0.2%)	1 (0.04%)	9 (0.24%)	9 (0.09%)
*Proteus* spp.	3 (0.08%)	15 (0.54%)	24 (0.64%)	42 (0.4%)
**Total**	**3**,**991 (100%)**	**2**,**788 (100%)**	**3**,**738 (100%)**	**10**,**508 (100%)**

* Counts for NASM (non-*aureus* staphylococci and mammaliicocci) include both genus-only and genus-plus-species identifications

**Table 2:** Total number of isolates for each *Enterococcus* species with their respective average MALDI-TOF MS log score

Species	Isolates with log score > 2.0 (%)	Average Log score	Isolates with log score 1.7–1.99 (%)	Average Log score
*E. saccharolyticus*	112 (53.33%)	2.16	76 (68.47%)	1.86
*E. faecium*	62 (29.52%)	2.32	15 (13.51%)	1.83
*E. cecorum*	12 (5.71%)	2.17	6 (5.41%)	1.93
*E. faecalis*	6 (2.86%)	2.30	9 (8.11%)	1.82
*E. casselliflavus*	6 (2.86%)	2.16	-	-
*E. hirae*	3 (1.43%)	2.32	3 (2.7%)	1.84
*E. devriesei*	3 (1.43%)	2.21	-	-
*E. canintestini*	1 (0.48%)	2.33	1 (0.9%)	1.95
*E. columbae*	-	-	1 (0.9%)	1.88
*E. durans*	1 (0.48%)	2.39	-	-
*E. glivus*	1 (0.48%)	2.36	-	-
*E. italicus*	1 (0.48%)	2.08	-	-
*E. malodoratus*	1 (0.48%)	2.02	-	-
*E. villorum*	1 (0.48%)	2.36	-	-
**Total**	**210 (100%)**	**2.22**	**111 (100%)**	**1**,**86**

* For 24 isolates, log scores were not found in the database: *E. cecorum* (1), *E. faecalis* (2), *E. faecium* (17), and *E. saccharolyticus* (4)

The species was not identified in 13 of 358 of the total enterococci isolates: three from MSC samples, eight from MC samples, and two from HS samples. Table 3 describes the *Enterococcus* species distribution across the different sample types. A total of 345 *Enterococcus* isolates were identified at the species level. Overall, *E. saccharolyticus* was the most prevalent (55.65%;192), followed by *E. faecium* (27.25%; 94) and *E. cecorum* (5.51%; 19). *E. faecalis* was identified in 4.93% (17) of the samples. Particularly, *E. saccharolyticus* was the most frequently isolated species from both CM (66.67%, 124 out of 186) and SCM (48.18%, 53 out of 110) milk, whereas *E. faecium* was the most frequently found in HS samples (44.9%, 22 out of 49). *E. cecorum* represented 10.91% (12 out of 110) of all isolates in SCM samples and was the only species with a significantly higher frequency in SCM compared to other sample types (p-value = 0.0128). Several other *Enterococcus* species, including *E. casselliflavus*, *E. hirae*, *E. devriesei*, *E. canintestini*, *E. columbae*, *E. durans*, *E. gilvus*, *E. italicus*, *E. malodoratus*, and *E. villorum* were collectively less than 7% of the total isolates.

**Table 3:** Distribution of *Enterococcus* species across sample categories

Species	CM quarter milk	SCM quarter milk	HS composite milk	Total general
*E. saccharolyticus*	124 (66,67%)	53 (48,18%)	15 (30,61%)	192 (55,65%)
*E. faecium*	41 (22,04%)	31 (28,18%)	22 (44,9%)	94 (27,25%)
*E. cecorum*	6 (3,23%)	12 (10,91%)	1 (2,04%)	19 (5,51%)
*E. faecalis*	6 (3,23%)	5 (4,55%)	6 (12,24%)	17 (4,93%)
*E. casselliflavus*	2 (1,08%)	4 (3,64%)	-	6 (1,74%)
*E. hirae*	2 (1,08%)	2 (1,82%)	2 (4,08%)	6 (1,74%)
*E. devriesei*	2 (1,08%)	-	1 (2,04%)	3 (0,87%)
*E. canintestini*	-	1 (0,91%)	1 (2,04%)	2 (0,58%)
*E. columbae*	-	1 (0,91%)	-	1 (0,29%)
*E. durans*	1 (0,54%)	-	-	1 (0,29%)
*E. glivus*	-	1 (0,91%)	-	1 (0,29%)
*E. italicus*	-	-	1 (2,04%)	1 (0,29%)
*E. malodoratus*	1 (0,54%)	-	-	1 (0,29%)
*E. villorum*	1 (0,54%)	-	-	1 (0,29%)
**TOTAL**	**186 (100%)**	**110 (100%)**	**49 (100%)**	**345 (100%)**

## Discussion

This retrospective study on 21,864 milk samples from Northern Italy over one year provides the first description of *Enterococcus* spp. in bovine milk in Italy using MALDI-TOF MS. The identification of 14 distinct species, with *E. saccharolyticus* (55.65%), *E. faecium* (27.25%), and *E. cecorum* (5.51%) as the most prevalent ones, is in line with emerging data at worldwide level and highlights the role of advanced microbiological tools in providing further information on mastitis epidemiology.

The predominance of *E. saccharolyticus*, particularly in milk from CM cases (66.67%), is in line with a recent report from Brazil, where a prevalence of 62.4% was recorded for CM using MALDI-TOF MS (Guimarães et al. 2024). This contrasts with earlier studies relying on biochemical methods, which identified *E. faecalis* as a prominent species (Różańska et al. 2019; Gao et al. 2019). The discrepancy suggests that traditional approaches, such as API systems or VITEK 2, may have misclassified *E. saccharolyticus* as *E. faecalis* or other SSLO due to overlapping phenotypic traits (Werner et al. 2014; Cameron et al. 2016).

The high frequency of *E. saccharolyticus* in CM samples prompts further research on its pathogenicity, potentially linked to environmental abundance, adaptability, resistance, and presence of virulence traits such as biofilm formation capabilities (Rodrigues et al. 2022). However, data on the specific role of *E. saccharolyticus* in bovine IMI remain scarce, and limited AMR has been reported for this species, suggesting distinct ecological or clinical profiles compared to other enterococci (Paschoalini et al. 2023).

In contrast, *E. faecium* emerged as the leading species in HS milk (44.9%), consistent with its frequent isolation from BTM in Brazil (67.8%; (Guimarães et al. 2024). This pattern may reflect its ecological adaptability and persistence in farm environments, such as bedding and fecal matter. In-vitro studies from our group have shown the survival and multiplication ability of *E. faecium* in lime-conditioned bedding, with minimal growth inhibition compared to other udder pathogens (Fusar Poli et al. 2024). These recent findings are in line with its prominence in composite HS samples from healthier quarters, while its lower prevalence in CM (22.04%) and SCM (28.18%) may suggest a less important role in IMI. On the other hand, its well-documented multidrug resistance, exceeding 20% (Liu et al. 2024), which could represent a challenge for infection treatment. Overall, the lower *Enterococcus* spp. prevalence in HS (1.36%) compared to CM (4.86%) and SCM (4.05%) could be related to dilution in pooled samples or to the healthier status of udders sampled for herd survey compared to mastitis diagnostic purposes.

The significant association of *E. cecorum* with SCM was a notable finding and represents its first reported connection to bovine mastitis in Europe. *E. cecorum* was previously identified as an emerging pathogen in poultry, causing septicemia and locomotor disorders (Laurentie et al. 2023), and from healthy calves (Devriese et al. 1992). Its isolation from SCM cases may suggest a potential subclinical persistence in dairy cows, while its lower frequency in CM (3.23%) and HS (2.04%) could imply adaptation to chronic, low-grade infections, possibly facilitated by virulence traits like epithelial adhesion, as seen in poultry isolates (Laurentie et al. 2023). However, its pathogenicity in bovine udders has not been investigated yet. On the other hand, this finding shows the value of MALDI-TOF MS in refining mastitis epidemiology, as highlighted by studies like Nonnemann et al. (2019). The technique’s enhanced ability to accurately identify a diverse range of bacteria overcomes some limitations of traditional biochemical methods, potentially revealing the involvement of species previously neglected or misclassified. The role of *E. cecorum* in SCM will require further studies to confirm its relevance and understand its pathogenicity.

The observed species distribution highlights the complexity of managing enterococcal mastitis, as different species may require different approaches. While this study did not assess AMR, the known species-dependent variability in resistance profiles reported elsewhere, such as higher resistance often found in *E. faecium* and *E. faecalis* compared to *E. saccharolyticus* (Gao et al. 2019; Paschoalini et al. 2023), shows the practical importance of the species-level identification achieved using MALDI-TOF MS. This could influence empirical treatment strategies for mastitis caused by this now-recognized prevalent pathogen, potentially allowing for more specific therapeutic approaches. This detailed epidemiological information is important for guiding future research on species-specific pathogenicity and resistance patterns, ultimately leading to more specific control strategies and prudent antimicrobial use (Ruegg 2022).

This study provides the first detailed report using MALDI-TOF MS to characterize *Enterococcus* species distribution within the context of bovine mastitis and herd health in Northern Italy. Our findings are representative of the region’s prevalent dairy systems, primarily involving Holstein-Friesian cows housed in free-stall barns. Understanding the specific enterococcal species prevalent under these European management and environmental conditions is important, as factors such as local climate, bedding materials, and antibiotic stewardship practices can significantly shape microbial ecology compared to other regions globally.

Potential study limitations should be acknowledged. Firstly, while MALDI-TOF MS is a powerful identification tool, the absence of routine molecular confirmation (e.g., PCR, sequencing) for all isolates carries a minor risk of misclassification, particularly concerning very closely related or rarer species like some identified here. Furthermore, 13 *Enterococcus* spp. isolates could not be identified to the species level. While this small proportion is unlikely to significantly impact the overall species trends observed, it highlights the need for updated databases and the potential utility of complementary molecular techniques for characterizing rare or novel isolates. Secondly, the retrospective analysis relied on samples submitted for routine diagnostics, which might not fully represent the complete epidemiological picture compared to a structured prospective sampling design.

## Conclusion

MALDI-TOF MS provided a detailed and reliable picture of enterococcal mastitis epidemiology, revealing an unexpected predominance of *E. saccharolyticus* in bovine mastitis compared to the previously reported *E. faecalis*, and indicating the relevance of emerging species as *E. cecorum*. Further investigations into pathogenicity, resistance patterns, strain specificities, and ecological drivers are expected to leverage the high reliability of this technology to enable a better understanding and optimization of enterococcal mastitis control.

## Data Availability

Data will be made available upon reasonable request.
